# NDM-5-carried outer membrane vesicles impair the efficacy of antibiotics against bacterial infections

**DOI:** 10.1128/aac.01805-24

**Published:** 2025-04-14

**Authors:** Lin Li, Yanfang Zhang, Liangyun Weng, Qianyu Ji, Feng Gao, Shuo Yang, Linran Fu, Yiming Gao, Xuan Ma, Mengying Zhang, Qingjun Xu, Yongning Wu, Shaoqi Qu

**Affiliations:** 1 Animal-Derived Food Safety Innovation Team, College of Veterinary Medicine, Anhui Agricultural University 605541 , Hefei, Anhui, China; 2 Research Unit of Food Safety, Chinese Academy of Medical Sciences (No. 2019RU014), NHC Key Laboratory of Food Safety Risk Assessment, China National Center for Food Safety Risk Assessment (CFSA) 442518, Beijing, China; University of Fribourg, Fribourg, Switzerland

**Keywords:** β-lactamase, outer membrane vesicle, *Enterobacteriaceae*, antibiotic

## Abstract

The intensifying use of antimicrobials in the rapidly growing livestock industry has heightened concerns over the proliferation of antibiotic resistance, particularly among *Enterobacteriaceae* producing β-lactamase. Elucidating the role of β-lactamase could unlock novel strategies to combat drug-resistant *Enterobacteriaceae* in livestock and poultry farming. Outer membrane vesicles (OMVs) produced by gram-negative bacteria have the ability to encapsulate and transport components derived from their parental bacteria. This raises the intriguing possibility that OMVs from drug-resistant bacteria could harbor drug-resistance enzymes, thereby conferring protection to susceptible bacteria against antibiotics. Here, we successfully extracted OMVs from New Delhi metallo-β-lactamase-5 (NDM-5)-expressing *Escherichia coli* and confirmed that these vesicles indeed carry NDM-5 protein. Furthermore, bacterial protection assays showed that these OMVs could cause sensitive bacteria treated with meropenem to restore growth activity, and the degradation of meropenem by the OMVs was verified using high-performance liquid chromatography. Lastly, the survival rate of the OMVs intervention group was significantly lower than that of the drug-treated group in a *Galleria mellonella* larvae infection model, validating the protective effect of these OMVs on sensitive bacteria and increasing their tolerance to meropenem. These findings illustrate that OMVs can serve as vehicles for resistance-related factors, thereby promoting antibiotic tolerance in susceptible bacteria.

## INTRODUCTION

The discovery of antibiotics has been a major advancement in medicine and has significantly reduced mortality rates. However, the widespread and excessive use of antibiotics in disease management has led to the emergence of bacterial antimicrobial resistance. The World Health Organization has identified the increasing prevalence of antibiotic resistance as a critical factor in the occurrence of severe, life-threatening infections (1). Carbapenems, often deemed the “last line of defense” against drug-resistant bacteria, are now facing significant challenges due to the emergence of carbapenemases, which have severely compromised their effectiveness (2). Despite carbapenems not being used in food animals, carbapenemase-carrying *Enterobacteriaceae* bacteria have been detected in animal populations worldwide, including farmed animals, companion animals, and wildlife (3). The problem of drug resistance in *Escherichia coli* of animal origin is becoming more and more prominent. In China, the average number of *E. coli* drug resistance genes carried doubled (from 8.05 to 16.85) in the 50 years from the 1970s to 2019, posing a serious threat to the livestock farming industry (4). A survey conducted on a chicken farm revealed that 55.8% of both chickens and environmental samples were contaminated with blaNDM-positive bacteria (5). Genes encoding carbapenemases include *Klebsiella pneumoniae* carbapenemase (KPC), imipenemase, Verona integron-encoded metallo-β-lactamase, and New Delhi metallo-β-lactamase (NDM), with blaNDM being the most prevalent carbapenemase gene found in *E. coli* in China (6, 7). The rapid spread of NDM within and between bacterial populations is primarily facilitated by highly transmissible mobile genetic elements (6, 8–10). This enables sensitive bacteria to persist in environments with β-lactam antibiotics. However, the role of NDM in the survival of *E. coli* sensitive to β-lactam antibiotics, when carried by other pathogens, remains unclear.

The outer membrane vesicles (OMVs) are nano-sized proteoliposomes released from the outer membrane of gram-negative bacteria, which are also the key carriers of material transportation (11–13). The lumen of OMVs has the capability to carry substances such as proteins and genes from bacteria. The unique membrane structure of OMVs not only shields the proteins from degradation by hydrolytic enzymes but also facilitates their membrane-binding function during transport, thereby aiding in the recognition and binding of receptor cells (14–16). Previous studies have shown that OMVs play a critical role in the transport of substances, including virulence factors (e.g., Shiga toxin, enterohemorrhagic *E. coli* hemolysin, flagellin, and cholera toxin) (17, 18) and drug resistance-associated factors (e.g., blaOXA-232, KPC) (19–21). Furthermore, they function as drug carriers and immune adjuvants in antigen and drug delivery and possess the capacity to act as decoys, bind, and degrade antibiotics (22, 23). While previous studies have demonstrated that OMVs from NDM-1-producing bacteria can facilitate the survival of susceptible strains (24), it remains unclear whether OMVs derived from different types of β-lactamase-positive *E. coli,* such as NDM-5-producing strains, exhibit similar effects in the presence of β-lactam antibiotics.

In this work, we aimed to investigate the influence of OMVs derived from NDM-5-expressing *E. coli* (N-OMVs) on the susceptibility of *E. coli* ATCC 25922 to meropenem (MEM). Our investigation encompassed an *in vitro* bacterial protection assay, high-performance liquid chromatography (HPLC) analysis, and *Galleria mellonella* larval infection assay. Our findings revealed that N-OMVs possess the capability to confer protection to susceptible *E. coli* in the presence of meropenem, thereby enabling normal growth to resume. Furthermore, the results of proteomics and enzyme-linked immunosorbent assay (ELISA) showed that N-OMVs indeed carry the NDM-5 enzyme. Therefore, we hypothesize that N-OMVs contribute to the development of antibiotic resistance in susceptible *E. coli*, providing new insights into bacterial resistance to antimicrobial agents, which will greatly benefit the treatment of bacterial infections in livestock farming.

## MATERIALS AND METHODS

### Bacterial strains

In this study, we utilized *E. coli* ATCC 25922 and NDM-5-expressing *E. coli* to isolate OMVs. The *E. coli* ATCC 25922 strain lacks drug resistance, serving as a control in the experiments. NDM-5-expressing *E. coli* was isolated from clinical samples and stored in our laboratory. Bacterial cultures were propagated in Luria-Bertani (LB) medium at 37°C with shaking at 200 rpm.

### Isolation and quantification of OMVs

The method for extracting OMVs was performed as follows: a single colony was selected and cultured in LB medium overnight, then the culture was expanded until reaching the late stage. The culture was then centrifuged at 5,000 × *g* for 15 min, and the supernatant was retained. This step was repeated once to eliminate the bacterial precipitate. The supernatant was then passed through 0.45 µm and 0.22 µm filter membranes in sequence using a vacuum filter. Next, the supernatant was concentrated 10-fold using a 100 kDa ultrafiltration tube (Millipore) and filtered again with a 0.22 µm filter membrane. The resulting sample was subjected to ultracentrifugation at 200,000 × *g*, 4°C for 2 h using an angle rotor (Ti 70, Beckman Coulter, USA) to obtain a crude extract of OMVs, and the OMVs were stored at −80°C.

The purification of OMVs was achieved using a density gradient centrifugation method. Optiprep (Sigma) density buffers with various concentration gradients were prepared and layered in ultracentrifuge tubes sequentially. The crude extract of OMVs was layered on top of the density layers and ultracentrifuged for 12 h at 150,000 × *g*, 4°C using a horizontal rotor (SW 41 Ti, Beckman Coulter, USA). One milliliter of each gradient was collected for Western blot (WB) detection. The density layer rich in OMVs was then centrifuged at 200,000 × *g* for 1 h at 4°C, and the resulting pellet was resuspended in phosphate buffered saline (PBS). Subsequently, OMVs were treated with DNase I and proteinase K to remove proteins and DNA in the OMVs environment.

### Characterization of OMVs

The protein concentration was determined using the bicinchoninic acid assay (BCA) method, following the manufacturer’s instructions for the BCA protein quantification kit (SparkJade, China). The corresponding BCA working solution was prepared and used in 96-well plates, with the protein content measured as OMVs content by an enzyme labeling instrument after incubation at 37°C for 30 min in the incubator.

OMVs were detected using nanoparticle tracking analysis (NTA, Particle Metrix, Meerbusch, Germany). Simultaneously, the trajectory of the Brownian motion of the OMVs nanoparticles was tracked to calculate the root mean square displacement of the nanoparticles per unit time interval, and the nanoparticle size distribution map was derived from the NTA result.

Purified OMVs were taken up in 20 µL on a carbon film copper mesh and left at room temperature for approximately 1 min. Once the samples were in a semi-dry state, they were negatively stained using uranium acetate staining solution, and after drying, they were observed by a JEM 1200 EX transmission electron microscope (TEM).

### EDTA assay

Equal volumes of N-OMVs were added to two separate portions of trypticase soy broth, one containing PBS and the other with 20 µL of 0.5 M EDTA solution. A sterile paper disc, impregnated with 10 µg of imipenem, was then placed in each of the mixtures, which were subsequently incubated at 37°C for 4 h. *E. coli* was cultured on Mueller-Hinton agar (MHA) plates. After incubation, the paper disc containing imipenem was removed from the solution and placed on the MHA plate inoculated with *E. coli*. The plate was inverted and incubated at 37°C for 18–24 h. The diameter of the resulting inhibition zone was then measured to assess the antimicrobial activity.

### NDM-5 detection by ELISA

An ELISA kit (Shanghai Keshun Technology Co., Ltd.) was used to detect the presence of NDM-5 enzyme in the samples. Bacterial cells were collected in a centrifuge tube, and the supernatant was discarded. To each bacterial pellet (corresponding to 10^8^ CFU), 1 mL of extraction buffer was added. The bacterial suspension was sonicated at 20% power for 3 s with 10 s intervals, repeated 30 times. The lysate was then incubated in a boiling water bath for 10 min, followed by centrifugation at 10,000 × *g* for 10 min at room temperature. The supernatant was collected, cooled, and analyzed according to the ELISA kit instructions. The lysis buffer was added to the OMV resuspension to disrupt its structure, ensuring the release of encapsulated proteins. The mixture was then incubated at 4°C for 30 min. The experiments were performed according to the ELISA kit instructions. The absorbance at 450 nm (OD_450_) was measured, and a sample was considered positive if the OD_450_ value exceeded the average optical density (OD) of the negative control plus 0.15.

### Polymerase chain reaction assay

Bacterial genomic DNA was extracted using a genomic DNA extraction kit (Bioengineering, Shanghai, China). Following the method of Rumbo et al. (25). OMV extracts were treated with proteinase K and DNase I to prevent contamination of the genome outside OMVs, and the OMVs were lysed with 0.1% Triton X-100 to release the DNA, which was then detected by polymerase chain reaction (PCR). The following primers were designed and used to amplify the NDM-5 gene: forward, 5′-TCAGCGCAGCTTGTCGGGCC-3′ and reverse, 5′-ATGGAATTGCCCAATATTATGCACC-3′.

### Sodium dodecyl sulfate polyacrylamide gel electrophoresis assay

Five micrograms of crude extract of OMVs was mixed with a loading buffer in a 4:1 volume ratio. Then we heated it in a metal bath at 100°C for 10 min to denature the mixture. We applied it to a 15% sodium dodecyl sulfate polyacrylamide gel electrophoresis (SDS-PAGE) gel (Sangon Biotech, China) using a steady pressure of 10 V for 30 min, then 80 V for 15 min, and finally 120 V for 1 h. Afterward, we removed it from the gel box. The gel was stained with Coomassie brilliant blue (Sangon Biotech, China) to observe the protein distribution of the crude extract of OMVs.

### WB assay

Proteins were separated using the SDS-PAGE procedure, and the separated proteins were transferred to a 0.45 µm polyvinylidene difluoride membrane at 380 mA for 40 min. The membrane was blocked with a rapid blocking solution (Beyotime Biotechnology, China). A polyclonal anti-OmpA antibody (Anhui Key Laboratory of Veterinary Pathobiology and Epidemic Disease Prevention and Control, China) was then diluted to a ratio of 1:5,000 and incubated overnight at 4°C. Following this, a secondary antibody coupled with horseradish peroxidase of goat anti-mouse IgG protein (Enogene, China) was applied to perform 1:10,000 dilution for immunoblotting. Finally, proteins were visualized by chemiluminescence using a Tanon 5200 imaging system.

### Proteomic analysis

The protein content of OMVs was quantified using the BCA protein assay kit (Bio-Rad, USA) following the manufacturer’s protocol. The extracted proteins were digested into peptide fragments using trypsin. The resulting peptides were analyzed by liquid chromatography-mass spectrometry. The raw MS data obtained for each sample were processed, identified, and quantified using MaxQuant software (version 1.6.14).

### *In vitro* protection assay

The protective effect of N-OMVs on *E. coli* ATCC 25922 was verified by incubating the initial concentration of 100 µg/mL of N-OMVs with meropenem-sensitive *E. coli* ATCC 25922 at 4 µg/mL of MEM in 96-well plates. Doubling dilution of N-OMVs to 1.56 µg/mL was used, with OMVs produced by *E. coli* ATCC 25922 (A-OMVs) as a negative control. The growth curves of *E. coli* ATCC 25922 were monitored by measuring the optical density at 600 nm (OD_600_) at 0, 8, 16, 24, and 36 h. Antibiotic susceptibility tests were used to confirm the protective effect of N-OMVs.

### Minimum inhibitory concentration

*E. coli* ATCC 25922 was cultured in LB broth until 10^8^ CFU/mL, then diluted 100 times to make a bacterial suspension. The bacterial suspension was mixed with 6.25 µg/mL N-OMVs (according to the result of the *in vitro* protection assay). After mixing, the bacteria solution was centrifuged at 5,000 × *g* for 10 min at 0, 4, 6, 12, and 24 h, respectively, and washed with PBS three times to remove OMVs. After adjusting the concentration of the bacterial solution to 10^6^ CFU/mL, the minimum inhibitory concentration (MIC) of meropenem was tested by the 96-well plate dilution method, and the experimental results were observed after culture at 37°C for 16 h.

### HPLC assay

The HPLC system and a C18 column were utilized for this experiment. Solvent A consisted of 0.1% aqueous ammonium formate (pH = 5.0), while solvent B was 100% acetonitrile. The injection volume was 10 µL with a flow rate of 1 mL/min, and elution conditions comprised 75% solution A and 25% solution B. The standard curve for meropenem was established at concentrations of 4, 8, 16, 32, and 64 µg/mL. After co-incubation of N-OMVs with MEM for 0, 2, 4, 8, 12, 16, 24, and 36 h, meropenem and N-OMVs were separated using a 0.5 mL 100 kDa ultrafiltration tube (Millipore), and the concentration of remaining meropenem was determined.

### *Galleria mellonella* infection model

The experimental approach to assess the protection of OMVs against *G. mellonella* infection was conducted as previously described (24). To lessen the impact on immune system function, a 4 h fasting period was implemented prior to initiating the *G. mellonella* infection model. Initially, the half lethal dose (LD50) of ATCC 25922 for *E. coli* larvae was determined. The therapeutic effect of MEM at different concentrations (low dose of 5 mg/kg [L-MEM], medium dose of 10 mg/kg [M-MEM], and high dose of 15 mg/kg [H-MEM]) on the *G. mellonella* infection model was then established, following the dosage guidelines for humans. Based on the LD50 assay, *E. coli* ATCC 25922 (2 × 10^8^ CFU/mL) was mixed with 0.5 µg/mL OMVs and injected at a volume of 20 µL into the left last limb of the *G. mellonella*, followed by MEM injection into the contralateral side after 1 h. The larvae were incubated at room temperature for 0, 12, 24, 36, and 48 h to monitor survival rates.

### Statistical analysis

Statistical analysis was performed using GraphPad Prism 9.0 (GraphPad Software, Inc.). A one-way analysis of variance test was used for all statistical analyses. All data were expressed as means ± SD.

## RESULTS

### Isolation and purification of OMVs

**Fig 1:**
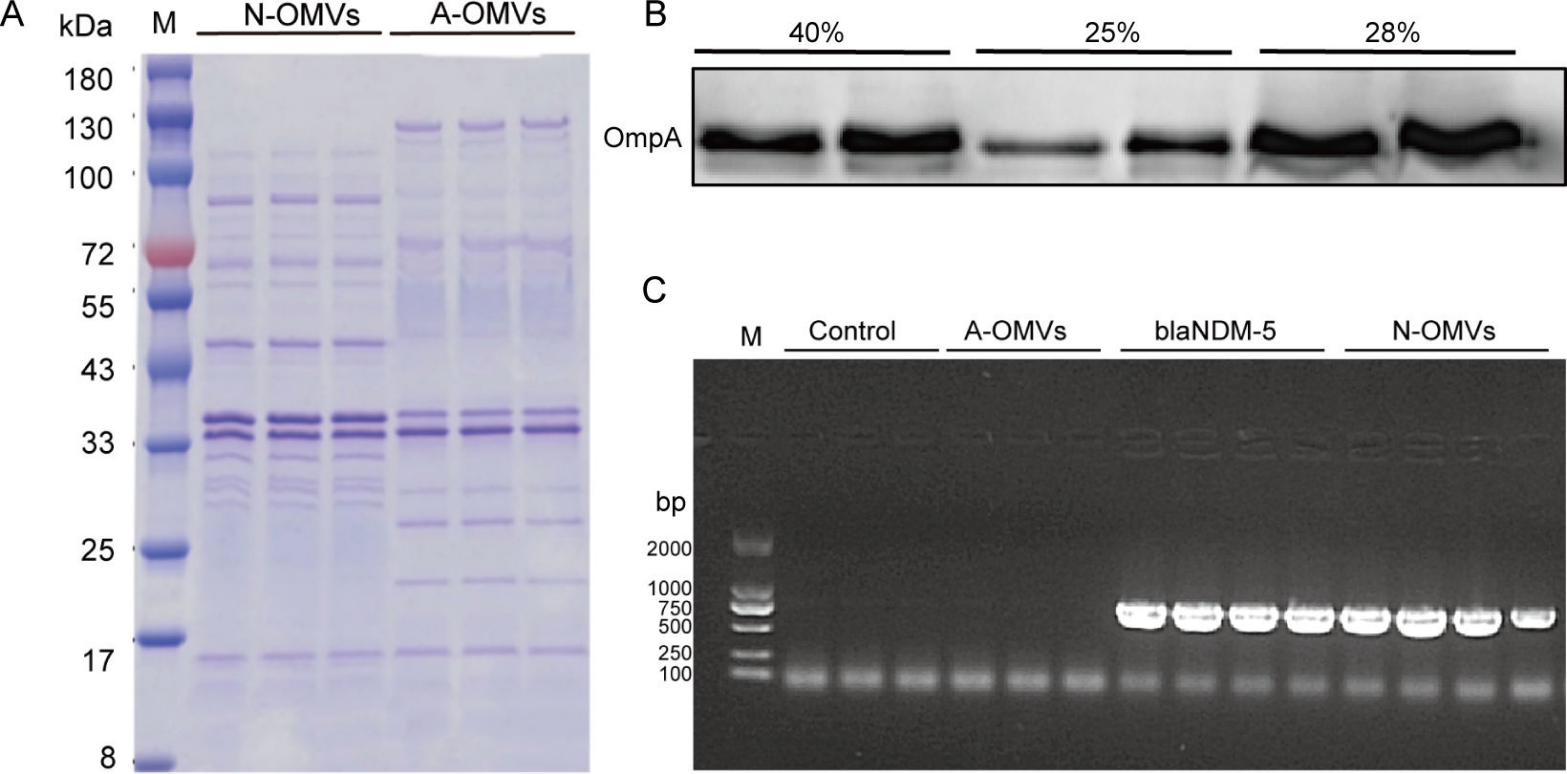
Identification of crude outer membrane vesicle (OMV) extracts. (A) SDS-PAGE for identification of proteins in N-OMVs and A-OMVs. (B) Western blotting confirmation of OMVs purified through density gradient centrifugation. The high content of OmpA protein in the outer membrane vesicles was used as a marker for WB identification. Equal protein concentrations and volumes from each gradient layer (25%, 28%, and 40%) were loaded into the gel wells. (C) PCR identification of the NDM-5 gene in N-OMVs. The NDM-5 gene fragment size was 813 bp.

The crude extracts of OMVs from NDM-5-expressing *E. coli* and *E. coli* ATCC 25922 were first obtained by ultracentrifugation and purified by density gradient centrifugation. To investigate the distribution of crude extracted liquid of OMVs in the Optiprep density gradient solution, we utilized SDS-PAGE and WB techniques for preliminary OMV identification. Coomassie brilliant blue staining revealed different OMV bands for both strains, but both strains showed bands corresponding to the size of OmpA (35 kDa), the most abundant protein in OMVs (Fig. 1A). For the WB assay, we aimed to add proteins of the same concentration and volume from individual gradient layers (25%, 28%, and 40%) to the gel wells. To achieve this, we concentrated the 3.6 mL 28% and 40% density gradient layers to 100–200 µL using 500 µL, 100 kDa ultrafiltration tubes (Millipore, USA). We used a polyclonal antibody against OmpA as the primary antibody and a horseradish peroxidase-labeled IgG antibody as the secondary antibody. Based on WB results, the 25%–40% Optiprep density layers all showed significant enrichment (Fig. 1B), but samples from the 28% and 40% Optiprep concentration layers were concentrated 20-fold before loading to match the protein concentration in the 25% density layer. Therefore, we ultimately selected the 25% Optiprep density layer recovery solution for OMV purification. These results suggest that our OMV extraction was successful.

### Characterization of OMVs

**Fig 2:**
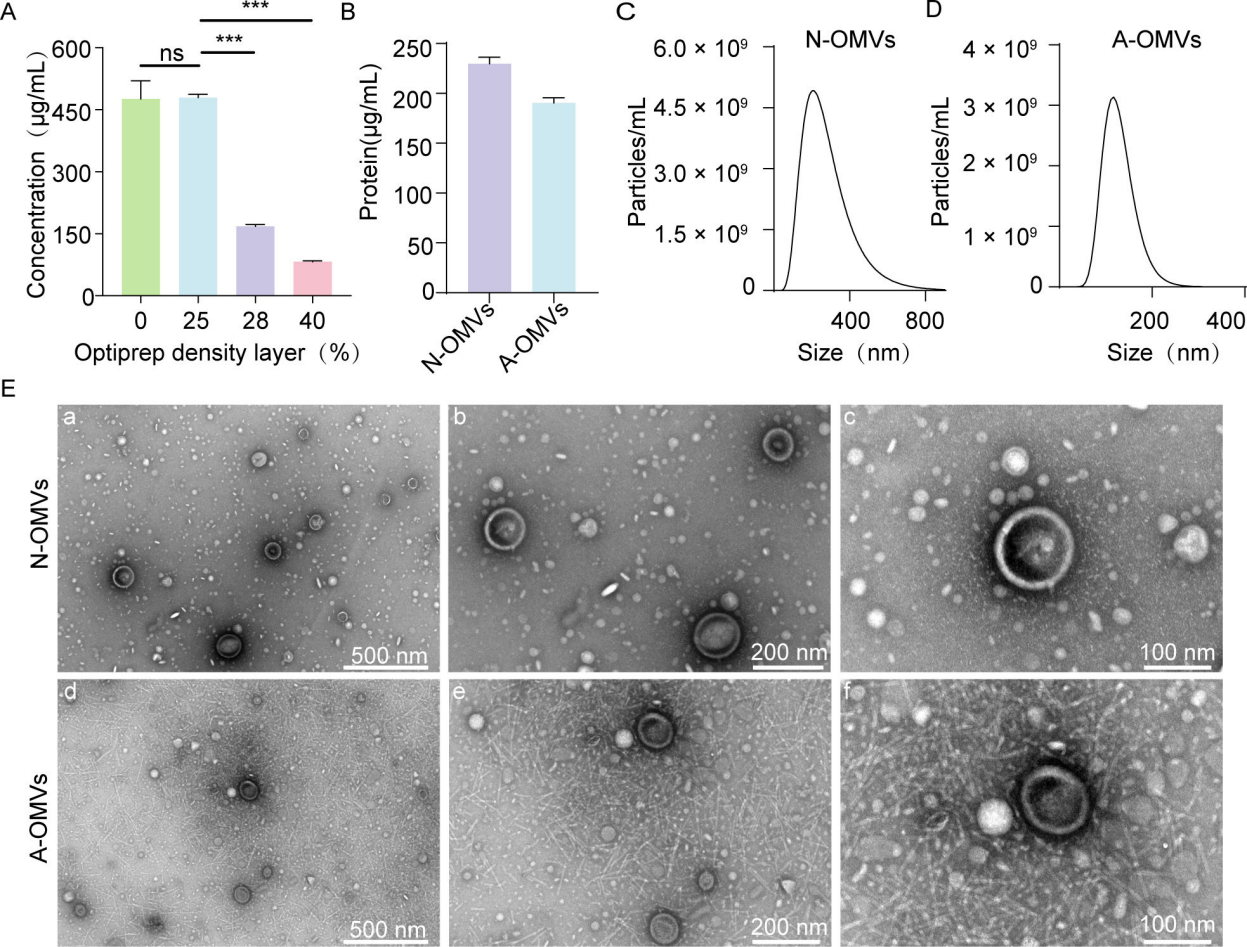
Characterization of OMVs. (**A**) Protein concentrations in the 0%, 25%, 28%, and 40% Optiprep density layers, measured using the BCA protein assay following density gradient centrifugation (*n* = 3). (**B**) Concentration of purified OMVs. Subsequent to purifying OMVs in the 25% Optiprep density gradient layer using ultracentrifugation, the concentrations of N-OMVs and A-OMVs were assessed using the BCA protein concentration assay kit (*n* = 3). (**C** and **D**) Nanoparticle size of OMVs based on nanoparticle tracking analysis (*n* = 3). (**E**) Representative TEM images of OMVs. ***P < 0.001.

After density gradient centrifugation, the protein concentrations in the 25%, 28%, and 40% Optiprep density layers were determined using the BCA protein assay. The 25% Optiprep density layer exhibited a high protein concentration (Fig. 2A). The liquid then underwent OMVs recovery, resulting in concentrations of 229.63 µg/mL for N-OMVs and 190.33 µg/mL for A-OMVs (Fig. 2B). To determine the particle size of the OMVs, NTA was employed. This revealed average diameters of 386.3 nm for N-OMVs and 187.0 nm for A-OMVs. A comparison showed that N-OMVs had a larger average diameter (Fig. 2C and D). This size difference could be due to the varied strain sources and cargo compositions of the OMVs. TEM imaging further showed the purified OMVs to be round or oval, varying in size, and with a complete double-layer membrane structure (Fig. 2E). The electron microscope image of A-OMVs showed partial flagella and/or fimbriae. The presence of flagella and/or fimbriae is unlikely to significantly interfere with the functional assays conducted in this study, due to the particle concentrations of both OMV types remaining within the same order of magnitude at equivalent protein levels.

### N-OMVs contained NDM-5 enzyme

We conducted additional experiments to identify the presence of β-lactamase in OMVs; we performed a Nitrocefin assay. The chromogenic results showed that the color response from yellow to red was produced under N-OMVs treatment (Fig. S1A), indicating that OMVs carried lactamase. To determine whether the β-lactamases in N-OMVs are metallo-β-lactamases, we utilized EDTA, a known metallo-β-lactamase inhibitor. The results showed that β-lactamase activity was inhibited by EDTA in OMVs (Fig. S2), indicating the presence of a metallo-β-lactamase (MBL) in the OMVs. Subsequently, we determined by ELISA that the MBL was an NDM-5 enzyme (Fig. S3), and these experiments proved that N-OMVs contained NDM-5 enzyme.

### Proteomic analysis of OMVs

**Fig 3:**
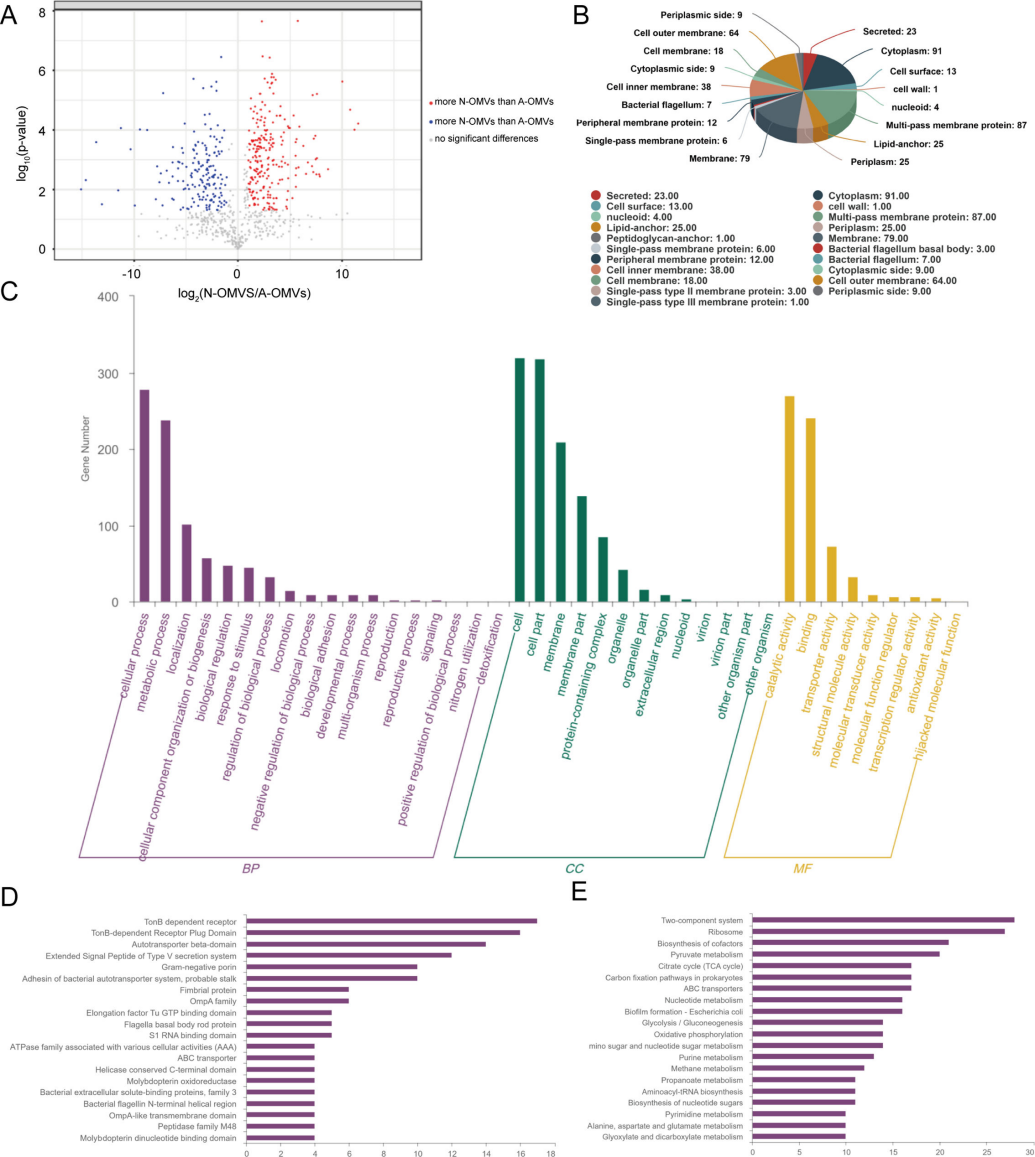
Proteomic analysis of OMVs. (**A**) A volcano plot of significantly differentially expressed proteins of N-OMVs vs A-OMVs. (**B**) Subcellular localization of differentially expressed proteins of N-OMVs versus A-OMVs. (**C**) GO annotation statistics plots of the differentially expressed proteins. (**D**) Field enrichment analysis plots of significantly differentially expressed proteins. (**E**) Plot of KEGG pathway annotation statistics for differentially expressed proteins.

To analyze proteins with differential expression between N-OMVs and A-OMVs, the experimental data were differentially screened. A total of 405 differentially expressed proteins were screened between N-OMVs and A-OMVs (Fig. 3A). The subcellular localization of all differentially expressed proteins was analyzed by CELLO, and a total of 21 subcellular localizations were identified (Fig. 3B). Gene ontology (GO) function annotation analysis was performed on 405 differentially expressed proteins, including 18 biological processes, 13 cell components, and 9 molecular functions (Fig. 3C). The domain prediction software interproscan was used to predict the domain of differentially expressed proteins. The differential expression of proteins is mainly concentrated in TonB-dependent receptors, TonB-dependent receptor insertions domain, autotransporter β-domain, autotransporter test domain, and V-type secretion system signal expanding peptides (Fig. 3D). The Kyoto Encyclopedia of Genes and Genomes (KEGG) pathway database was used to perform functional analysis of N-OMVs and A-OMVs proteome, and the results showed that a total of 309 differentially expressed proteins were annotated (Fig. 3E). Furthermore, we listed eight of the differentially significant proteins in the table, and NDM-5 protein is one of them (Table S2).

### N-OMVs promote the resistance of *E. coli* to MEM

**Fig 4:**
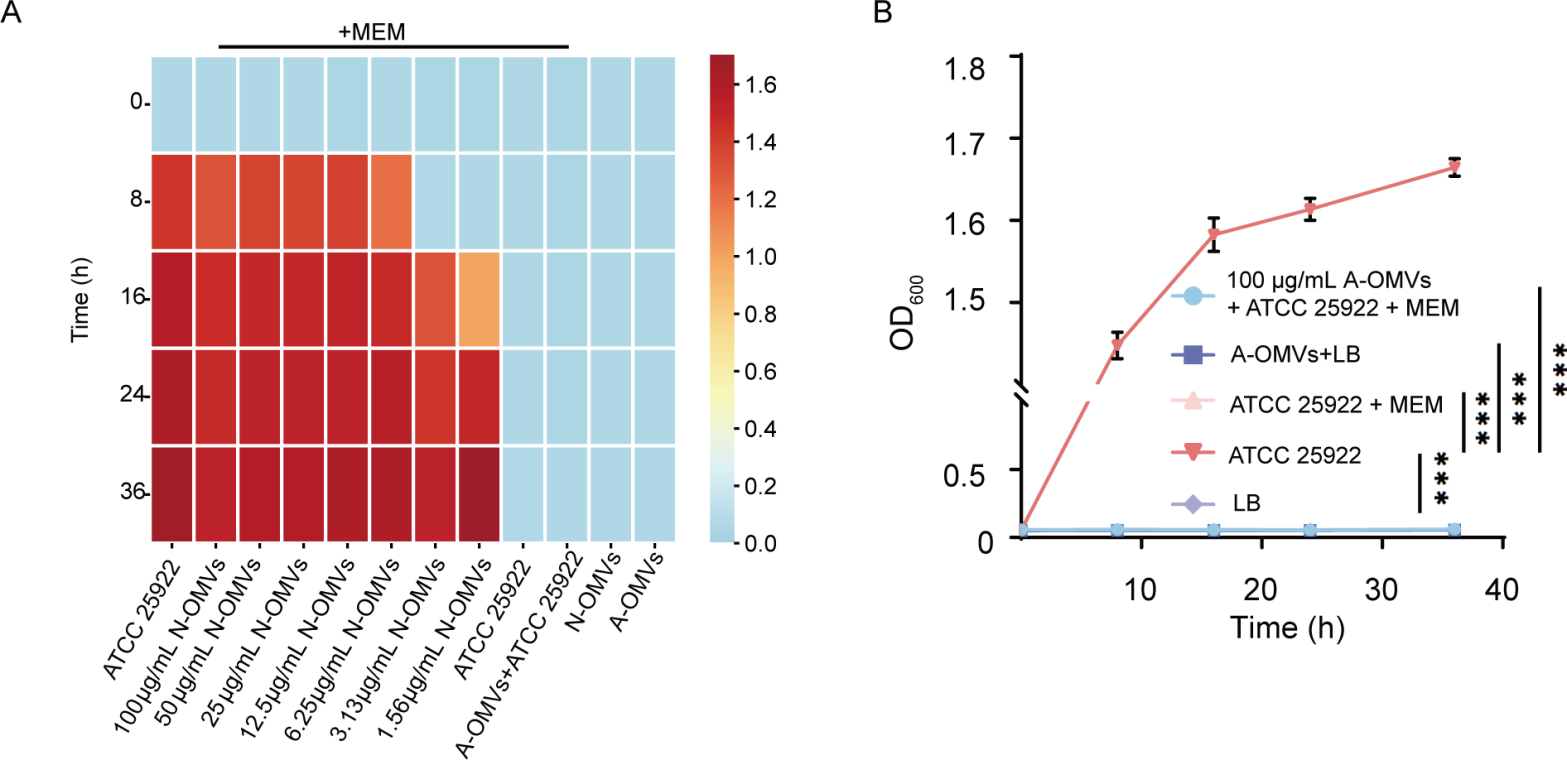
N-OMVs enhance the resistance of *E. coli* to MEM. (**A**) Heatmap illustrating the protective effect of N-OMVs on *E. coli*. Each row represents the incubation time (0, 8, 16, 24, and 36 h). Column 1: *E. coli*. Columns 2–8: *E. coli* in MEM environment treated with varying concentrations of OMVs. Column 9: *E. coli* co-incubated with MEM. Column 10: *E. coli* treated with 100 µg/mL A-OMVs and MEM. Column 11: N-OMVs. Column 12: A-OMVs. (**B**) A-OMVs did not exhibit a protective effect on *E. coli*. Darker colors indicate higher bacterial concentrations. Data are represented as mean ± SD (*n* = 3). Statistical significance was determined by an unpaired two-tailed *t-*test. ***P < 0.001.

To evaluate the ability of NDM-5-expressing *E. coli*-derived N-OMVs to protect *E. coli* ATCC 25922 against MEM killing, MIC and minimum bactericidal concentration (MBC) experiments were conducted. We found that the MIC and MBC of MEM against *E. coli* ATCC 25922 were 0.5 µg/mL and 1 µg/mL, respectively. Furthermore, co-incubation with 4 µg/mL of MEM for 48 h demonstrated that N-OMVs enabled *E. coli* ATCC 25922 to thrive in MEM at four times the MBC, with a concentration-dependent protective effect (Fig. S4). Furthermore, low concentrations (3.13, 1.56 µg/mL) of N-OMVs initially showed no protective effect on *E. coli* within 8 h, but resulted in restored growth at 16 h, indicating a time-dependent protective effect (Fig. 4A). Additionally, we found that 6.25 µg/mL of N-OMVs exhibited the same protective ability as 100 µg/mL, indicating the effective protection of N-OMVs. Importantly, A-OMVs did not demonstrate the protective effect (Fig. 4B), suggesting that NDM-5-carried N-OMVs hydrolyze meropenem to protect sensitive strains of *E. coli* from lethal concentrations of antibiotics, rather than due to physical adsorption of vesicles. Lastly, we found that the protective effect observed in the sensitive strain is temporary and only lasts for the duration of exposure to the OMVs carrying the NDM-5 enzyme. After the co-incubated OMVs were removed, the MIC value was within the sensitive range, and the protective effect disappeared (Table S1). This indicates that the resistance is due to the presence of the NDM-5 enzyme in the OMVs, which degrades the antibiotic, rather than a permanent genetic change.

### N-OMVs mediate the MEM degradation

**Fig 5:**
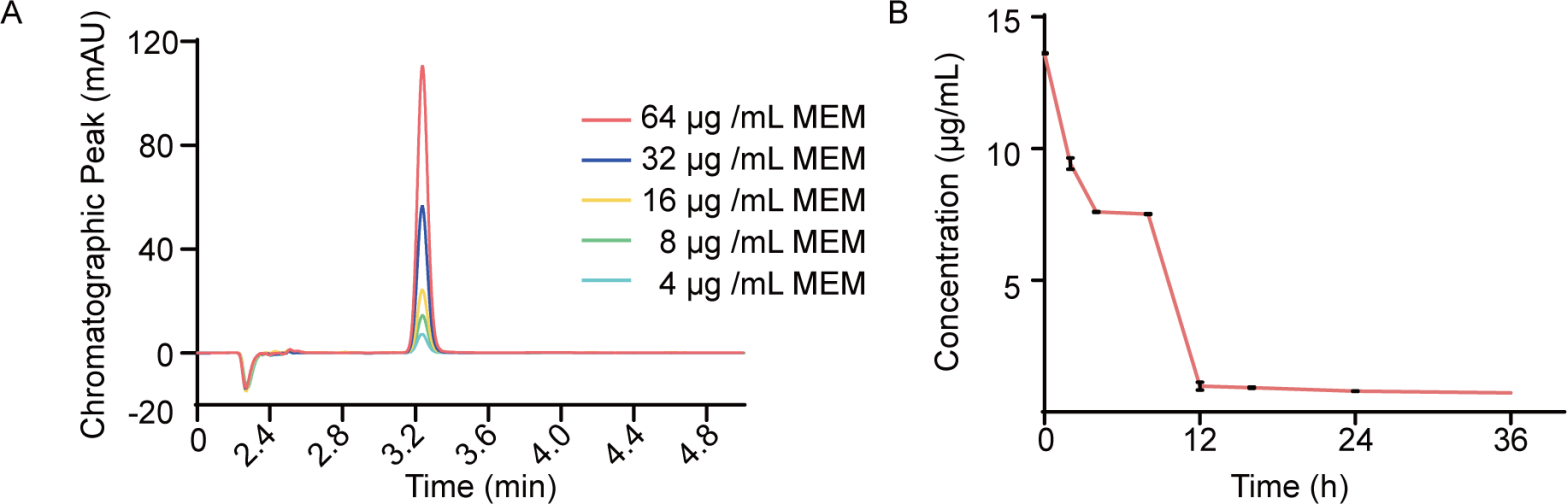
N-OMVs have the ability to degrade MEM *in vitro*. (A) The concentration of MEM was detected using HPLC. MEM samples were prepared at concentrations of 4, 8, 16, 32, and 64 µg/mL. The increase in peak height was concentration-dependent with the concentration of MEM drugs. (B) Incubation of 12.5 µg/mL N-OMVs and 16 µg/mL MEM for 0, 2, 4, 8, 12, 16, 24, and 36 h, followed by the removal of OMVs using an ultrafiltration tube. The remaining concentration of MEM in the filtrate was detected by HPLC.

To evaluate the degradation rate of MEM by N-OMVs, MEM concentrations were measured using HPLC at 0, 2, 4, 8, 12, 16, 24, and 36 h after co-incubation with N-OMVs. MEM at concentrations of 0, 8, 16, 32, and 64 µg/mL were selected to establish a standardized working curve (Fig. S1B). The height of the chromatographic peak is directly proportional to the concentration of MEM (Fig. 5A), indicating that it can be used for the quantification of MEM in the sample. Based on the results of bacterial protection and HPLC tests (Fig. S1C and D), the initial MEM concentration was 16 µg/mL. The results showed that 6.25 µg/mL N-OMVs effectively degraded MEM in a short time, and 52.5% of the MEM was hydrolyzed by N-OMVs at 4 h (Fig. 5B). In addition, the hydrolysis of MEM by N-OMVs reached a “plateau” at 4–8 h, and the degradation rate basically stopped. At 12 h, the degradation of MEM by N-OMVs decreased from 16 μg/mL to 0.98 μg/mL, with a degradation rate of 93.9% (Fig. 5B). These findings are consistent with the results of bacterial protection experiments (Fig. 4), suggesting that N-OMVs effectively improve the survival of susceptible bacteria by rapidly degrading MEM.

### N-OMVs protect *E. coli* from bactericidal imipenem treatment in *G. mellonella*

**Fig 6:**
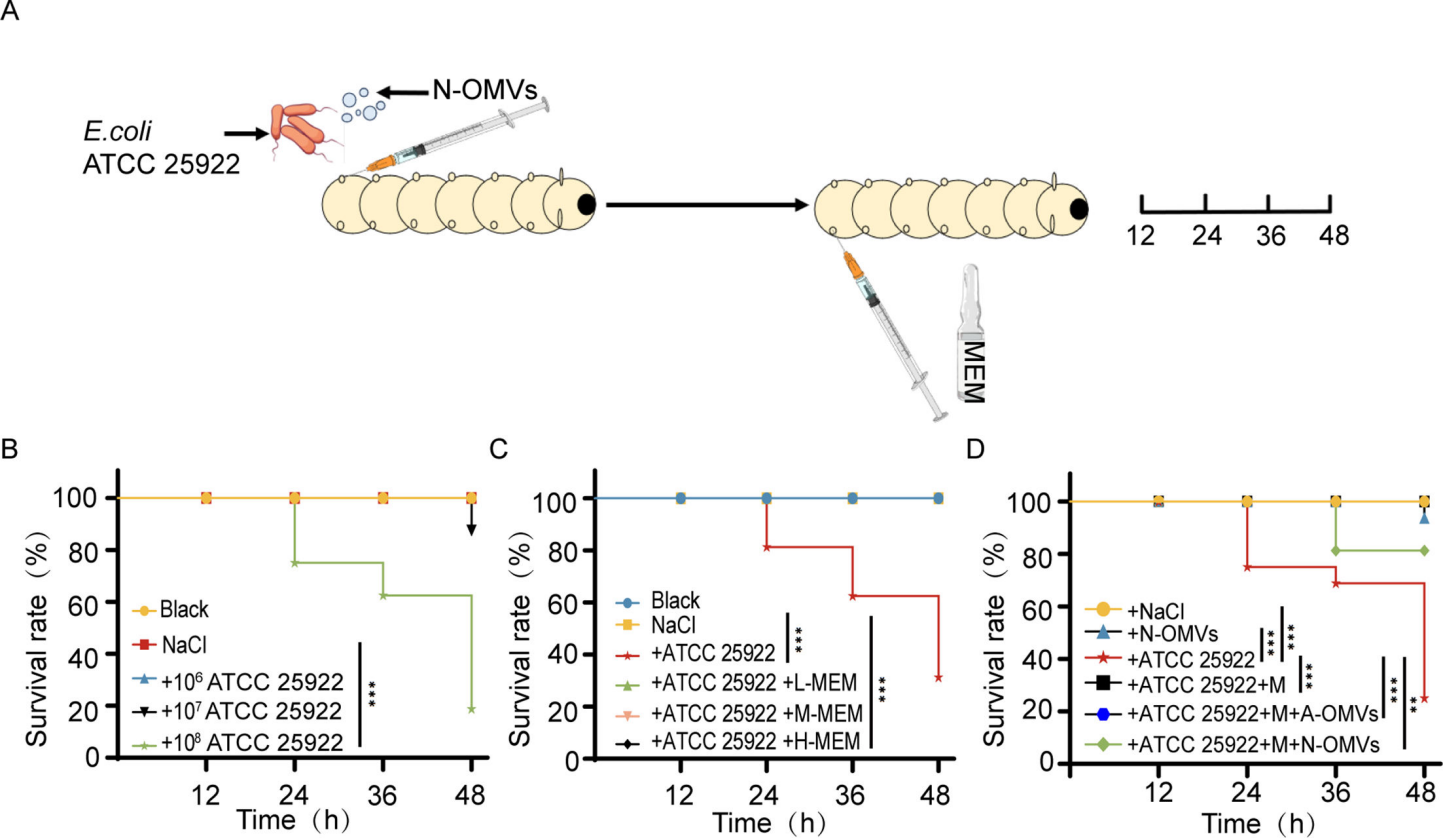
N-OMVs invalidate MEM in *G. mellonella* to promote *E. coli* infection. (**A**) Illustration of the *G. mellonella* infection model. The bacteria and N-OMVs were injected into the left last ventral foot of the larvae (300 ± 50 mg), and MEM was injected into the opposite side 1 h later. Larvae survival rates (*n* = 15) were recorded over 48 h. (**B**) Determination of the lethal dose of *E. coli* ATCC 25922 in *G. mellonella*. Larvae were injected with *E. coli* at 10^6^, 10^7^, and 10^8^ CFU/mL, with the dose resulting in 80% mortality within 48 h selected for experiments. (**C**) Concentration screening of MEM. MEM at low dose (L-MEM, 5 mg/kg), medium dose (M-MEM, 10 mg/kg), and high dose (H-MEM, 15 mg/kg) were set to screen the drug dosage for the treatment group (*n* = 15). (**D**) OMVs reduced the efficacy of MEM against *E. coli*. Larvae survival rates at 0, 12, 24, 36, and 48 h post-injection were monitored to assess the protective effect of N-OMVs on *E. coli in vivo* (*n* = 15). Statistical significance was determined by the Kaplan-Meier test. **P < 0.01, ***P < 0.001.

To evaluate the *in vivo* protective effect of N-OMVs on *E. coli* ATCC 25922, a *G. mellonella* infection model was established (Fig. 6A). The initial experiment revealed that a concentration of 1 × 10^8^ CFU/mL of *E. coli* ATCC 25922 resulted in an 80% mortality rate in *G. mellonella* larvae within 48 h (Fig. 6B), thus this concentration was selected as the lethal dose. MEM at low dose (L-MEM, 5 mg/kg), medium dose (M-MEM, 10 mg/kg), and high dose (H-MEM, 15 mg/kg) were utilized to assess the clearance rate of *E. coli* ATCC 25922 in *G. mellonella* larvae model. The results showed that each concentration of MEM effectively eliminated *E. coli* ATCC 25922 and preserved 100% larval survival (Fig. 6C), indicating the high efficacy of MEM. However, after the intervention of N-OMVs, the survival rate of the infected *G. mellonella* treated with MEM was reduced to 81.25%, indicating that N-OMVs significantly reduced the therapeutic effect of MEM. Collectively, these results demonstrated that N-OMVs severely impair the efficacy of antibiotics to aggravate the severity of bacterial infections.

## DISCUSSION

The use of antibiotics in livestock and poultry farming is crucial to raising healthy and productive animals. However, the widespread and diverse use of antimicrobial drugs has contributed to the escalating issue of drug resistance in animals. *E. coli*, a common zoonotic foodborne bacterium, is known for its pathogenicity and can be effectively treated with antibiotics. Nevertheless, the emergence of carbapenemase-producing *Enterobacteriaceae* poses a significant public health threat globally (26). The emergence of new patterns of microbial resistance poses a serious challenge to the effective prevention and control of drug-resistant bacteria.

*E. coli* is resistant to β-lactam antibiotics by producing β-lactamase which exists in the periplasmic space of bacterial pathogens (27). Among these enzymes, NDM is notable for its broad-spectrum activity against β-lactam antibiotics. However, these free hydrolases are vulnerable to environmental factors in *in vivo* settings leading to their damage and inactivation (24). OMVs serve as the ingenious “long-distance transport carriers of bacteria,” effectively safeguarding the “cargo” within their unique structure (19). Leveraging the unique properties of OMVs and their prowess in transporting bacterial endogenous substances, it has been unveiled that OMVs can transport antibiotic hydrolases derived from drug-resistant bacteria, thereby shielding these enzymes from environmental inactivation (28). It has been definitively proven that NDM interacts with the bacterial membrane with a stable, well-defined conformational interaction and that membrane interaction helps NDM secretion of this enzyme in OMVs (29, 30). This same phenomenon has been observed in *Pseudomonas aeruginosa* and *Salmonella* spp. (31–33). Our study found and verified that N-OMVs can carry the blaNDM-5 gene and NDM-5 enzyme (Fig. 1C; Fig. S3).

OMVs play a role in defense against antibiotics when bacteria are stimulated by antibiotics. Because the structure of OMVs is similar to the bacterial outer membrane, they can be used as a “dummy target” to bind with drugs and divert the drug’s attack on bacteria (22). Studies have shown that *Escherichia coli* OMVs can bind to polymyxin, reduce polymyxin contact with bacteria, and protect bacteria from drugs (34). OMVs can also work in concert with bacterial efflux mechanisms, and Huang et al. (35) found that *Acinetobacter baumannii (A. bauxnii)* could use efflux to expel the antibiotics in the periplasmic space in the OMVs. The OMVs secreted by bacteria can not only load the drugs in the bacteria but also carry the enzymes of hydrolyzing drugs to make the drugs degrade and deactivate (36). Our work demonstrated that OMVs derived from NDM-5-expressing *E. coli* protected sensitive *E. coli* ATCC 25922 from being exterminated by MEM, providing strong evidence that antibiotic hydrolase-carrying OMVs present a mechanism for bolstering bacterial resistance to antibiotics. However, the mechanism by which OMVs interact with antibiotics is not fully understood, and further experiments are needed to confirm it.

### Conclusion

Our work provides additional evidence that outer membrane vesicles derived from NDM-5-expressing *E. coli* are capable of carrying and transporting NDM-5 enzyme and enhancing the tolerance of susceptible bacteria. This suggests that in a mixed bacterial community, susceptible bacteria may be protected by N-OMV against antibiotic killing. The transportation of antibiotic hydrolases via OMVs represents a novel mechanism for bacteria to acquire resistance to antibiotics. Furthermore, the overuse of antibiotics contributes to the synthesis of antibiotic hydrolases, which are subsequently packaged into OMVs and released by bacteria, thereby conferring susceptible bacteria with the ability to counter antibiotic killing both *in vivo* and *in vitro*.

## SUPPLEMENTAL MATERIAL

Supplemental material aac.01805-24-s0001.docx N-OMVs contain NDM-5 enzyme, protective against *E. coli*.

ASM does not own the copyrights to Supplemental Material that may be linked to, or accessed through, an article. The authors have granted ASM a non-exclusive, world-wide license to publish the Supplemental Material files. Please contact the corresponding author directly for reuse.
