# Supplementary material for: NDM-5-carried outer membrane vesicles impair the efficacy of antibiotics against bacterial infections
Source: Antimicrob Agents Chemother. 2025 Apr 14;69(5):e01805-24. doi: 10.1128/aac.01805-24 (PMC12057343; doi:10.1128/aac.01805-24)
Supplement: Supplemental material — N-OMVs contain NDM-5 enzyme, protective against E. coli. [file aac.01805-24-s0001.docx]

Supplementary Materials for

**NDM-5-carried outer membrane vesicles impair the efficacy of antibiotics against bacterial infections**

Lin Li^a,#^, Yanfang Zhang ^a,#^, Liangyun Weng ^a^, Qianyu Ji ^a^, Feng Gao ^a^, Shuo Yang ^a^, Linran Fu ^a^, Yiming Gao ^a^, Xuan Ma ^a^, Mengying Zhang ^a^, Qingjun Xu ^a^, Yongning Wu ^a,b,*^, Shaoqi Qu ^a,*^

^a^Animal-Derived Food Safety Innovation Team, College of Animal Science and Technology, Anhui Agricultural University, Hefei 230036, China

^b^Research Unit of Food Safety, Chinese Academy of Medical Sciences (No. 2019RU014), NHC Key Laboratory of Food Safety Risk Assessment, China National Center for Food Safety Risk Assessment (CFSA), Beijing 100022, China.

^#^These authors contributed equally to this work.

*Corresponding author: Y. Wu, Email: [wuyongning@cfsa.net.cn](mailto:wuyongning@cfsa.net.cn); S. Qu: sqq@ahau.edu.cn.

Supplementary Table

**Table S1.**  The MIC value changed after different times of N-OMV co-incubation.

| Incubation time (h) | 0 | 4 | 6 | 12 | 24 |
| --- | --- | --- | --- | --- | --- |
| MIC (μg/mL) | 0.5 | 0..5 | 0.5 | 0.5 | 0.5 |

**Table S2.**  Some significant differential proteins between A-OMV and N-OMV

| Protein No. | Gene name | Protein name | P value |
| --- | --- | --- | --- |
| A0A346CG54 | *blaNDM-5* | Beta-lactamase NDM-5 | 0.00165 |
| A0A192CKP5 | *ompA* | Outer membrane protein A | 0.00364 |
| A0A0V9LPK0 | *fadL* | Long-chain fatty acid transport protein | 0.00310 |
| A0A376VUA9 | *traT* | TraT complement resistance protein | 0.00008 |
| Q1R6W8 | *ygiW* | Bacterial OB-fold domain-containing protein | 0.00260 |
| Q8CVW1 | *ompC* | Outer membrane protein C | 0.00135 |
| W1F740 | *lolA* | Outer-membrane lipoprotein carrier protein | 0.02224 |
| P20966 | *fruA* | PTS system fructose-specific EIIBBC component | 0.00021 |

Supplementary figures


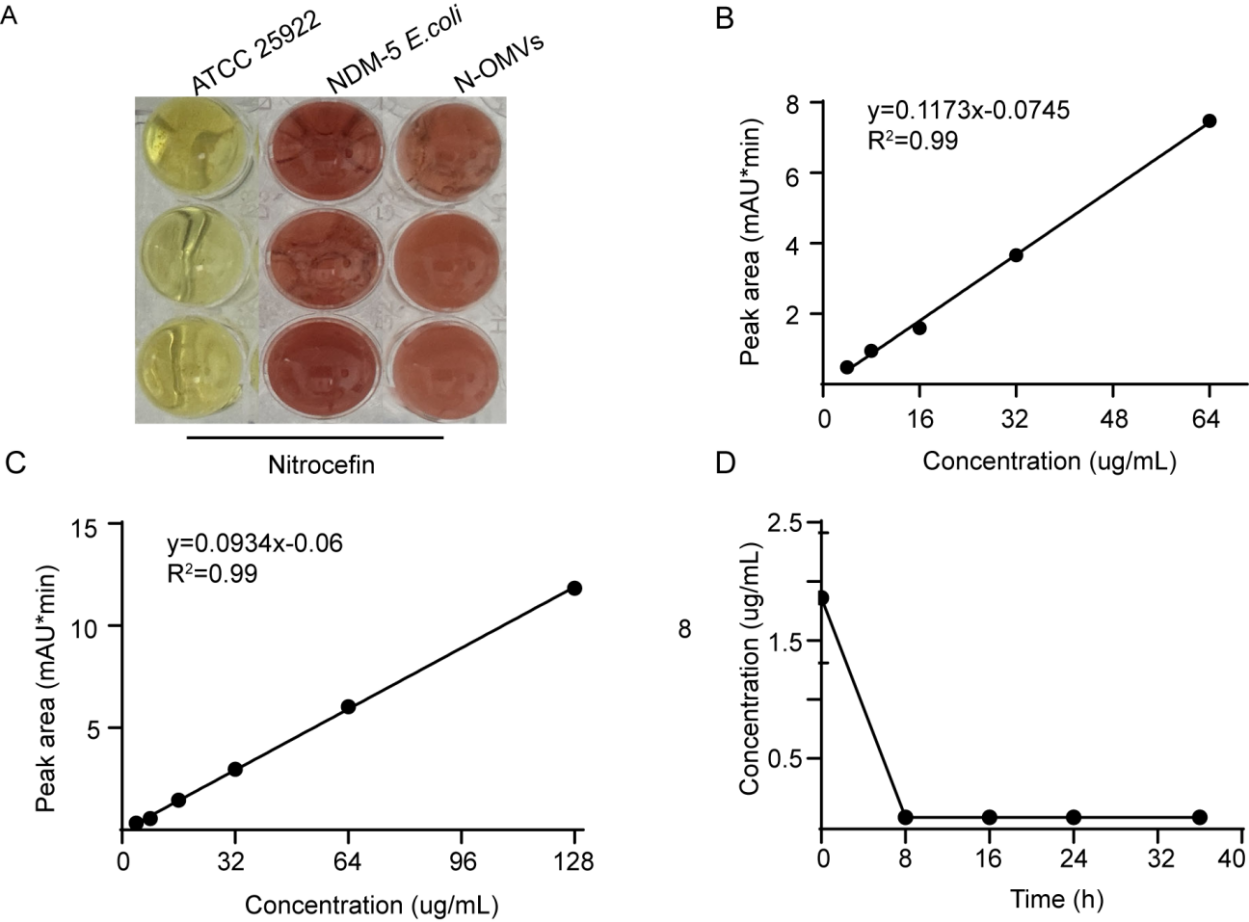


**Fig S1.**  Characterization of lactamase-carried OMVs.

(A) N-OMVs harbor beta-lactamase, which catalyzes the hydrolysis of nitrocefin. A 96-well plate was supplemented with a 1 mg/mL solution of cefnitrothiene. The experimental design included the following columns: Column 1: Negative control, containing the protein crude extract of ATCC 25922 (lacking beta-lactamase). Column 2: Positive control, with the addition of NDM-5 E. coli protein crude extract (harboring beta-lactamase). Column 3: N-OMVs (n=3 replicates).

(B) Establishment of a MEM standard working curve. This working curve is aligned with the standard curve obtained when 12.5 µg/mL of N-OMVs is incubated with 16 µg/mL of MEM, as depicted in Fig. 4A. MEM concentrations of 4, 8, 16, 32, and 64 µg/mL were prepared to construct HPLC standard operating curves (n=3 replicates).

(C) Establishment of a MEM standard working curve. The working curve corresponds to the standard curve generated when 12.5 µg/mL of N-OMVs is incubated with 4 µg/mL of MEM, as shown in Fig. S1D. MEM concentrations of 4, 8, 16, 32, 64,and 128 µg/mL were formulated to establish HPLC standard operating curves (n=3 replicates).

(D) Determination of the degradation effect of N-OMVs on MEM using HPLC. The residual MEM concentrations were analyzed by HPLC after incubating 12.5 µg/mL of N-OMVs with 4 µg/mL of MEM for 0, 8, 16, 24, and 36 hours (n=3 replicates).


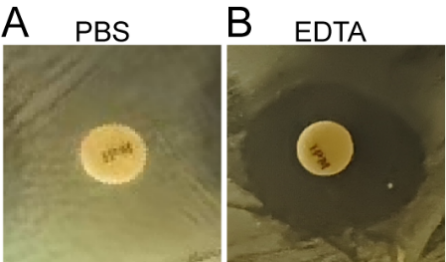


**Fig S2.**  EDTA inhibits the β-lactamase activity in OMVs.

The same amount of N-OMVs was added into Trypticase Soy Broth (TSB) broth with PBS (A) or with 20 µL of 0.5 M EDTA solution (B), and sterile paper containing 10 µg of imipenem was soaked in it, and incubated at 37°C for 4 h. *E. coli* ATCC25922 coated on Mueller-Hinton Agar (MHA) plate. Taking imipenem paper out of the broth, stick it on an MHA plate coated with *E. coli* ATCC25922, invert the plate, and incubate at 37°C for 18-24 h. Measure the diameter of the bacteriostatic circle.


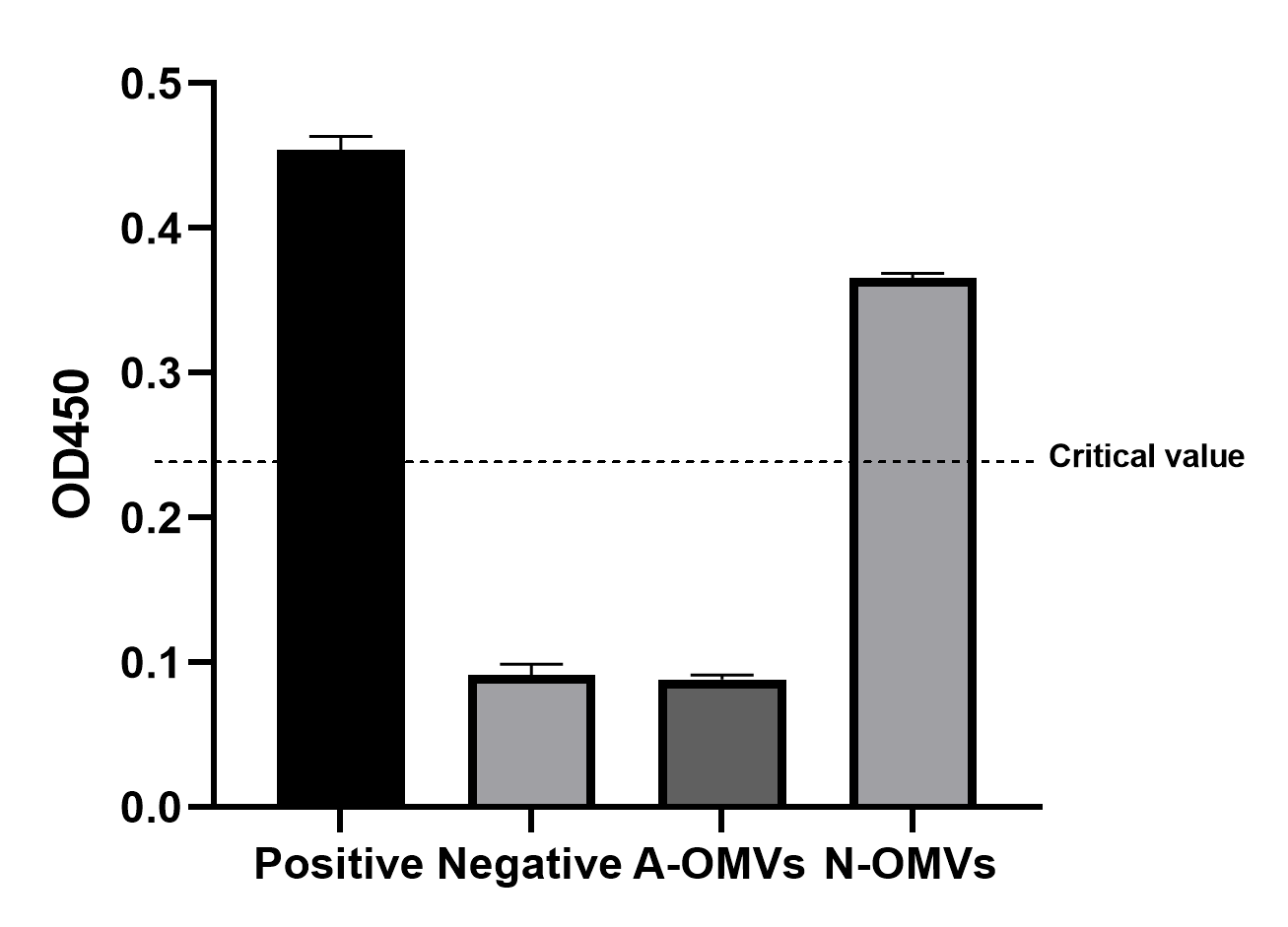


**Fig S3.**  Detection of NDM-5 in N-OMV by ELISA.

The presence of NDM-5 enzyme in N-OMV was qualitatively detected using an ELISA kit specific for Escherichia coli NDM-5. A sample was considered positive if its absorbance at 450 nm exceeded the critical value. "Positive" refers to NDM-5-positive Escherichia coli, "Negative" refers to double-distilled water, and the critical value is defined as the negative control absorbance plus 0.15.


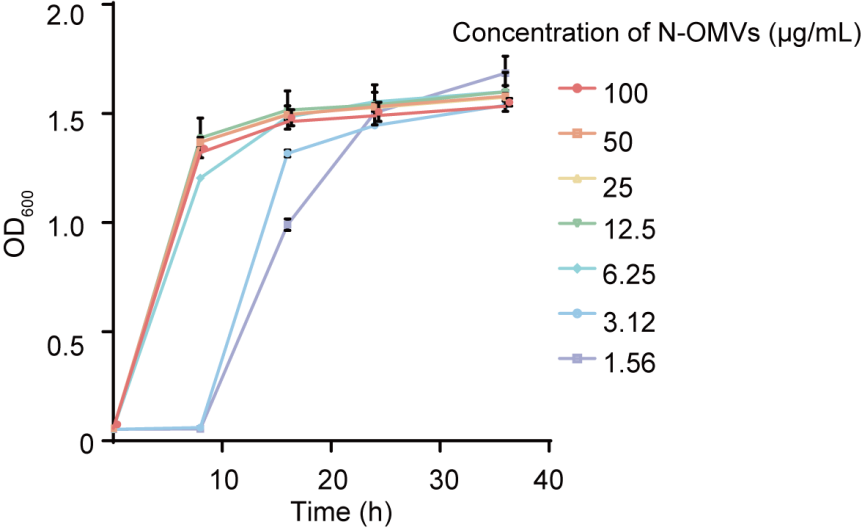


**Fig S4.** N-OMVs enhance the resistance of *E. coli* to MEM.

The protective capacity of N-OMVs was evaluated by monitoring the growth of *E. coli* in the presence of bactericidal concentrations of MEM. N-OMVs at a concentration of 100 µg/mL were diluted to 1.56 µg/mL in 96-well plates (n=3). The results showed that N-OMVs were effectively protected against *E. coli*.
